# Facilitators and barriers in managing older chronic heart failure patients in community health care centers: a qualitative study of medical personnel's perspectives using the socio-ecological model

**DOI:** 10.3389/frhs.2025.1483758

**Published:** 2025-04-24

**Authors:** Yan Lou, Min Zhang, Yun Zou, Le Zhao, Yangfan Chen, Yongzhen Qiu

**Affiliations:** ^1^ Department of Nursing, Hangzhou Normal University, Hangzhou, Zhejiang, China; ^2^ Department of Health College, Zhejiang Zhoushan Tourism and Health College, Zhoushan, Zhejiang, China; ^3^ Department of Nursing, Hangzhou Zhalongkou Street Community Health Service Center, Hangzhou, China; ^4^ Medical Affairs Department, Haining People's Hospital, Jiaxing, China; ^5^ Department of Cardiology, Lishui Central Hospital and Fifth Affiliated Hospital of Wenzhou Medical College, Lishui, Zhejiang, China

**Keywords:** community health care, chronic heart failure, chronic disease management, qualitative research, social ecology model

## Abstract

**Background:**

Community health care centers (CHCs) plays a crucial role in ensuring timely diagnosis and effective management of congestive chronic heart failure (CHF) in older patients. Understanding the current status of CHF management in CHCs can therefore be effective in reducing the disease burden of CHF.

**Objectives:**

This study evaluates the current state of CHF services in community healthcare facilities and identifies key facilitators and obstacles faced by medical personnel in China.

**Methods:**

This interpretive study applied the social ecological model (SEM) and used a semi-structured interview guide for data collection. Each interview lasted 45–60 min. Thematic analysis was used to analyze the data.

**Results:**

This study involved 30 participants. Facilitators and barriers were identified within the five domains of the SEM. (1) Individual level: medical staff lack knowledge and experience in CHF management while patients' need for greater health education. (2) Interpersonal level: insufficient support from the patients' family and lack of trust in CHCs and staff. (3) Organizational level: inadequate medical knowledge and training programs for medical staff, shortage of medical staff and limited teamwork and few health promotion channels. (4) Community level: Lack of regular screening and follow-up, medical equipment and an information technology-assisted monitoring system. (5) Public policy level: lack of policy support, funding subsidies, national guidelines adapted to the local context and low medical insurance reimbursement rate.

**Conclusion:**

There are many impediments to chronic disease management in the community, so it is vital to improve public understanding of CHF, as well as to improve the quality of community health equipment and services, to improve reciprocal referral mechanisms between hospitals and the community, and to develop policies on chronic disease management for CHF.

## 1 Introduction

Chronic heart failure (CHF) refers to a persistent state of heart failure due to myocardial damage caused by any reason such as myocardial infarction, cardiomyopathy, hemodynamic overload, inflammation, etc., resulting in changes in myocardial structure and function, and ultimately leading to ventricular failure. The blood pumping or filling function is low and cannot meet the body's needs. The main clinical manifestations are dyspnea, fatigue and fluid retention (1). There are more than 63.6 million patients worldwide, and there are 8.9 million patients in China alone (2, 3). Early symptoms of CHF may be atypical and difficult to detect, especially in older patients with other comorbidities such as hypertension, diabetes, renal failure, lung disease, and cardiac arrhythmias (4, 5). Missed diagnosis, untimely treatment, and incorrect management have led to the high prevalence and heavy disease burden of CHF (4, 5). In addition to these challenges, hospitals readmit about 25% of patients within 30 days of discharge due to disease recurrence, increasing both the physical and economic strain on patients and healthcare systems (6). CHF poses a significant threat to the health of older adults and therefore requires improved management and intervention.

Community Health Care centers (CHCs) play a crucial role in the management of chronic diseases (7, 8). They identify potential patients through early screening, conduct professional diagnosis and assessment, establish comprehensive health records, and implement the resident doctor contracting system, providing patients with comprehensive, systematic, continuous, and personalized management services for chronic diseases (9). At present, CHCs in China have provided chronic disease management services, but mainly for hypertension, diabetes and chronic obstructive pulmonary disease, and cannot meet the needs of CHF (10, 11). For patients with CHF, early diagnosis and effective management are essential (12, 13). There is a lack of integration and implementation of key services, such as outpatient clinics, routine disease screening, and follow-up care (14, 15). As a result, older CHF patients are still hospitalized and die at high rates. Therefore, effective community chronic disease management plays an important role in patients with CHF.

CHCs staff play an important role in the chronic disease management of CHF patients. They have direct contact with patients and have a good understanding of their conditions. They are also the implementers of policies, so they are also familiar with current policies. In order to improve the health status of patients with CHF, it is crucial to investigate the current status and potential challenges of chronic disease management in the community. Qualitative research can provide profound insights into the lived experiences and perspectives of CHCs staff implementing geriatric CHF services (16). This will reveal actual barriers and facilitators in current practice and inform the development of more effective community management measures.

Using the social-ecological model (SEM) as its theoretical foundation, this qualitative study explores community-based chronic disease management for older adults with CHF. SEM allows for an in-depth exploration of the factors that influence CHF management at multiple levels, including personal, interpersonal, organizational, community, and public policy. Identify facilitators and barriers to effective CHF management through these different levels (17). There are currently no studies applying this model to community-based management of CHF. To fill this gap, this study provides a comprehensive analysis of the current status of community-based chronic disease management. This research aims to establish a solid theoretical foundation to guide the development of more effective, integrated, and efficient programs, and eco-friendly CHF management strategies in community settings, leading to better health results for CHF patients.

## 2 Methods

### 2.1 Setting

This study was conducted in CHCs in Hangzhou, Jiaxing and Lishui in Zhejiang Province, China. This study investigated the chronic disease management practices in community hospitals in Zhejiang Province, which is well known for its advanced economic and medical services. Hangzhou, Jiaxing and Lishui represent the most advanced, medium and relatively weak areas in Zhejiang province respectively. Hangzhou has a population of 11.936 million and a population density of 719 people per square kilometer. It is the provincial capital city. This study selected two CHCs from Hangzhou, among which one community health care center in Shangcheng District of Hangzhou serves at least 70,000 patients every year, with general clinics and chronic disease management clinic, which is the benchmark among CHCs in Zhejiang Province. Two CHCs in Jiaxing serve 40,000 and 300,000 people respectively, which are representative in Jiaxing; One community health care center in Lishui participated in this study, and the CHCs serve more than 40,000 people per year. These CHCs all have integrated chronic disease service centers, and which provides chronic disease management, health education, and follow-up care for patients with chronic conditions such as heart failure. The variety in both city and countryside environments within these locations provided an extensive perspective on the execution and obstacles of managing chronic illnesses.

### 2.2 Study design and guiding framework

**Figure 1:**
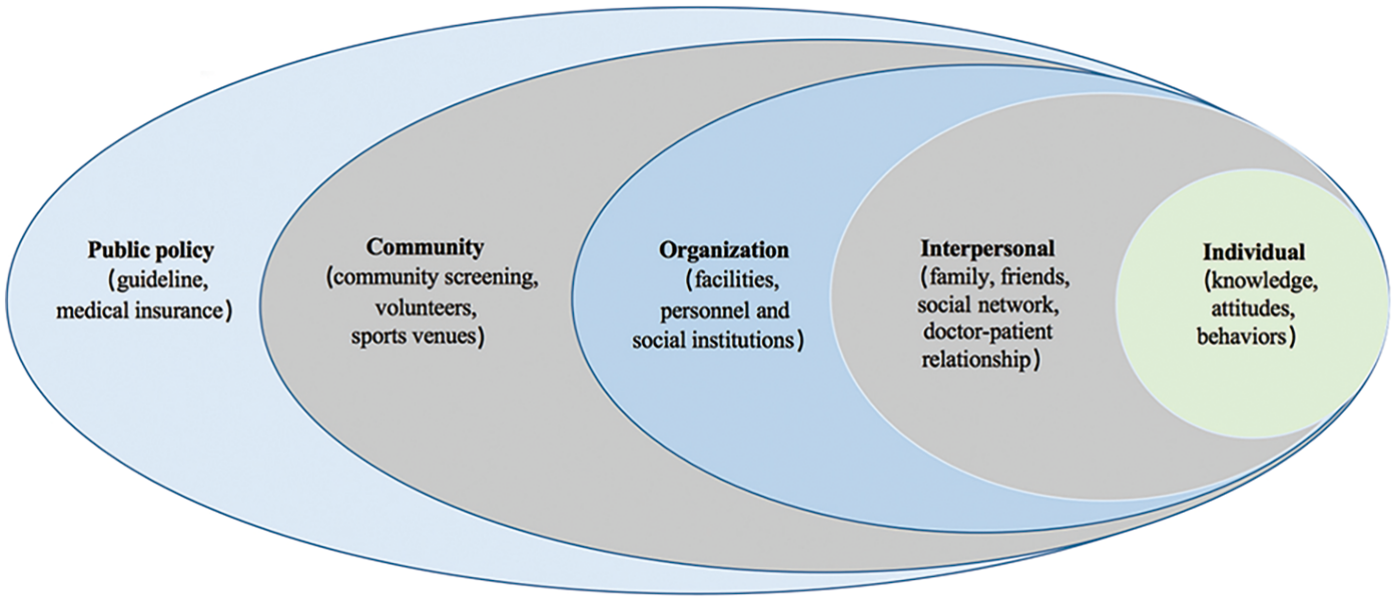
Five-level socio-ecological model.

In order to understand the current state of chronic disease management in CHF and the factors that facilitate or hinder its implementation, this study employed qualitative interviews with healthcare professionals from CHCs. As a guiding framework, the SEM encompasses five levels of influence: individual, interpersonal, organizational, community, and public policy (18). Patients' characteristics, knowledge, attitudes, and self-management abilities were examined at the individual level. Patient relationships with family members, friends, and healthcare providers were examined at the interpersonal level. Healthcare organizations were examined at the organizational level for their role in providing care and resources. At the community level, we assessed the broader community environment, including social networks and community resources. Finally, the public policy level evaluated the influence of policies and regulations on health behavior and healthcare practices (Figure 1). By applying the SEM, we conducted a comprehensive analysis that considered multiple levels of influence, allowing us to identify key facilitators and barriers across different social levels and understand their complex interplay.

### 2.3 Sampling

The study employed purposive and snowball sampling techniques to identify potential participants, ensuring a diverse representation of doctors and nurses in terms of age, years of experience, professional titles, and other relevant factors. Subsequently, exponential non-discriminative snowball sampling was utilized to enhance the recruitment process. A sensitive nature of the interviews and the potential for reidentification make it impossible for other researchers to replicate the results.

### 2.4 Recruitment

In July 2023, we approached 7 CHCs, and 5 agreed to participate which are in Hangzhou, Jiaxing, and Lishui. We established communication with the administrators of these health centers. These administrators, in turn, reached out to potential participants who fulfilled the necessary criteria. According to the paper published by Hennink and Kaiser in 2022, the number of studies in qualitative studies reaches 9–17 people, which can reach saturated (19). Considering that some personnel did not accept the interviews, We initially contacted a total of 35 participants. The author electronically transmitted a flyer, a participant information sheet, and a consent form to the interviewees, elucidating the study's objectives and the interview procedures.

Healthcare personnel meeting the specified criteria were deemed eligible for participation, including doctors, nurses, and managers in CHCs. Additional requirements included a minimum of five years of professional experience, fluency in Mandarin, willingness to participate, and the ability to provide informed consent. Subsequently, potential participants were contacted, and their qualifications were verified. The study was thoroughly explained, participant information sheets were distributed, and any pertinent inquiries were addressed.

In accordance with ethical guidelines, participants were provided with written informed consent and a mutually agreed upon interview schedule was established. Prior to the interviews, participants were provided with a comprehensive explanation of the study's objectives, methodologies, advantages, and potential impacts. The interviewer explicitly emphasized the voluntary nature of participation and assured participants that their provided information would remain confidential. As a token of appreciation, each participant received a gift valued at approximately $20 US. Ultimately, 30 participants met the criteria and completed the interviews. Interviews were arranged for all individuals who expressed interest in participating.

### 2.5 Data collection

After establishing contact with potential participants and ensuring their eligibility through rigorous screening, we proceeded with data collection via semi-structured face-to-face interviews. These interviews, guided by a theoretical framework based on existing literature and expert opinion, were aligned with the five-level SEM (Table 1). Each interview, conducted in Mandarin, lasted 45–60 min in the hospital's conference room. All interviews were recorded, ensuring anonymity for participants. Prior to each session, participants completed demographic questionnaires. Information saturation was achieved with the 30th interview.

**Table 1:** Interview schedule.

Does the community hospital carry out chronic disease management for chronic heart failure, and is it necessary?
What services does the Community Health Center provide for older patients with chronic heart failure?
What factors would facilitate the development of chronic heart failure management in community health care centers, and what benefits would these factors bring?
What difficulties are faced in managing older chronic heart failure in the community? (Including aspects such as patients, family members, medical personnel, medical equipment, and social environment)
What is the current referral situation in tertiary and community health care centers, and how can we improve the referral process for chronic heart failure patients?
What are the requirements for medical staff to carry out community chronic heart failure management, and is continuing education needed?
How can older chronic heart failure patients, family members, the community, and social media help in managing chronic heart failure in the older?
How should chronic disease management be conducted during infectious disease outbreaks (e.g., post-COVID-19)?

To balance content and methodological expertise, two researchers (one clinician scientist and one qualitative research expert) conducted the interviews. One led the interview while the other took notes and prompted further discussion as needed. Transcripts were reviewed by participants for feedback and potential corrections. We piloted the interview guide at Lishui Liandu and Hangzhou Shangcheng CHCs, refining questions for clarity. Recruitment continued until data richness adequately met the study objectives (19).

### 2.6 Data analysis

The data analysis was conducted following Braun and Clarke's 6-step thematic approach (16). The primary author thoroughly reviewed each transcript multiple times, taking into account the study objectives in the process of developing the codes. The data provided by the respondents were carefully examined for each concept. Data immersion was achieved through repeated readings of the transcripts and subsequent translation into English. The interviews, once transcribed, were analyzed using NVivo 12, which was utilized to import the transcriptions, field notes, and reflective diaries for analysis. Other team members can participate in data analysis using NVivo 12's collaboration features. They can jointly engage in coding and theme identification processes. Utilizing the consistency check tool ensures coding consistency among different researchers. A number of research team members reviewed the coded transcripts and collated data from code reports to identify broader patterns of meaning (e.g., themes). Our next step was to evaluate the themes against the data in order to develop a detailed analysis of each and to choose an informative name for each. Each transcript was refined and described until no new concepts were found in the remaining transcripts, ensuring saturation of themes. At least three ways were utilized to enhance rigor: (1) multiple investigators coded data and participated in analysis and interpretation; (2) multiple informants were interviewed; (3) collecting CHCs' protocols and documents. Having a diverse research team led to a more in-depth discussion and understanding of the conceptual content of the data.

During the analytic phase, all research team members participated in regular team meetings where disagreements were discussed openly and consensus reached. Deductive coding was employed at each of the five levels of the SEM to initially apply the SEM to the data. To facilitate the review of the initial concepts, a thematic mind map was constructed. Analytical triangulation was conducted by involving a second researcher to code and evaluate a subset of two information-rich, anonymized transcripts, thereby enhancing the dependability of the findings. The research team engaged in an analysis and discussion of the code lists, ultimately reaching a consensus on the interpretation of the study's findings. Furthermore, the broader research team contributed to a more comprehensive examination and interpretation of the data.

### 2.7 Ethics statement

The ethics statement underwent approval by the research ethics committee of the Lishui Central Hospital. Prior to the collection of data, informed written consent was acquired from all participants.

### 2.8 Rigor

The method outlined by Braun and Clarke's 6-step thematic was employed to establish rigor (20). To enhance the rigor, credibility, and applicability of the study, purposive sampling, reflective diaries, peer reports, thick transcripts, on-site notes, recordings, and memos were utilized. Additionally, extensive transcription, field notes, memoranda, and audio recordings were employed to further enhance the trustworthiness of the study.

### 2.9 Patient and public involvement statement

It was not designed, conducted, reported, or disseminated by patients or the public. Patients or the public were not involved because the study focused specifically on the perspectives of healthcare professionals and aimed to understand the systemic factors influencing chronic disease management from a provider's viewpoint.

## 3 Results

**Figure 2:**
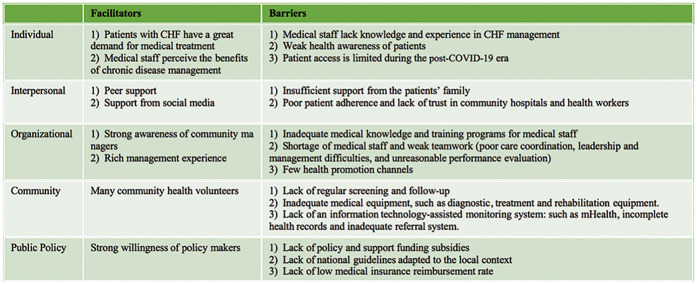
Barriers and facilitators in context of SEM.

A total of 30 participants were interviewed in this study, with a mean age of 42.7 ± 12.1 years. Their professional titles were distributed as follows: 3 junior, 21 intermediate, and 6 seniors. The mean duration of their professional experience was 9.8 ± 6.2 years. Most participants resided in urban areas (Table 2). Figure 2 illustrates the primary themes identified across different levels of the SEM, highlighting the factors that facilitate or impede the management of CHF as revealed through the interviews.

**Table 2:** Summary of participant demographics.

Characteristic number (%)	Variable	Participants, *n* = 30
Title		
	Primary	3
	Intermediate	21
	Senior	6
Years of experience		
	5–10	6
	11–20	15
	21–30	8
	Above 31	1
Highest academic education		
	Junior college	10
	Undergraduate	18
	Master	2
Age range		
	20–30	5
	31–40	11
	41–50	13
	Above 51	1
Region		
	Jiaxing	10
	Hangzhou	10
	Lishui	10
Designation		
	Community nurse	16
	Community doctor	14
Gender		
	Female	26
	Male	4
Positions	Manager	6
	Staff	24

### 3.1 Individual

#### 3.1.1 Facilitators

At the individual level, this study identified two key themes related to the enhancement of chronic disease management in CHF. These themes include the urgent need for better accessibility to care for CHF patients and the perceived benefits of community-based management for chronic diseases in older individuals, as recognized by healthcare professionals. Nearly all healthcare providers expressed their active involvement in the initiative, citing regular clinical interactions with CHF patients. They strongly supported the project, emphasizing its importance in contributing to public health and addressing the high prevalence of CHF in the country.

#### 3.1.2 Barriers

##### 3.1.2.1 Medical staff lack knowledge and experience in CHF management

In the management of CHF, medical staff commonly face issues related to insufficient knowledge and experience. Many healthcare professionals lack systematic training and practical experience in the diagnosis and management of CHF. For example, a community nurse stated, “I currently have limited understanding of chronic heart failure and lack specific training on this condition.” Additionally, some doctors and nurses report difficulties in implementing chronic disease management procedures, particularly in developing long-term treatment plans and monitoring disease progression. An internal medicine doctor noted, “Managing chronic heart failure requires ongoing tracking and adjustments, but we lack relevant training and support, leading to suboptimal management outcomes.”

##### 3.1.2.2 Limited health awareness of patients

CHF patient health literacy poses a significant challenge. One community physician expressed dissatisfaction with patient adherence: “Many patients reduce their medication dosage or stop taking it altogether due to side effects.” Another physician highlighted difficulties in persuading patients to reduce their salt intake, noting that deeply ingrained dietary habits make it challenging for patients to make necessary dietary changes even when aware of the health risks. Additionally, some patients hold traditional beliefs that illnesses should be treated with rest rather than physical activity. As one medical professional put it, “Certain individuals choose to endure illness rather than seek medical help, which always delays their treatment.”

##### 3.1.2.3 Patient access is limited during the post-COVID-19 era

Additionally, the availability of healthcare facilities during epidemics and other contagious diseases is limited. A medical professional mentioned, “During the ongoing phase of the epidemic and in the post-COVID-19 era, hospitals saw reduced patient attendance as people avoided exposure to infection, while many medical staff were engaged in outbreak response efforts.”

### 3.2 Interpersonal

#### 3.2.1 Facilitators

In the community, primary sources of support for patients include robust social media platforms and peer support groups. According to a county health director, an active social media presence allows patients to access a wealth of information about their condition and connect with others who have similar experiences. The director also highlights the significant role of support groups, noting that the success stories shared within these groups can greatly enhance patients' well-being and overall experience.

#### 3.2.2 Barriers

##### 3.2.2.1 Insufficient support from the patients' family

Several key interpersonal barriers hinder the effective management of CHF in community settings. First, limited family engagement is a major challenge. One community physician emphasized, “Family support and supervision are vital for managing chronic diseases. Family members spend the most time with the patient, have frequent contact, and play a crucial role in monitoring the patient. However, currently, family members are not providing the necessary support.”

##### 3.2.2.2 Poor patient adherence and lack of trust in CHCs and staff

Another significant barrier is the lack of comprehensive patient education initiatives. A nurse pointed out, “We offer limited health education related to chronic heart failure, such as lectures and other activities. These initiatives are infrequent and not well-integrated into our care protocols.” Moreover, patients' distrust of community healthcare services and poor compliance with treatment are substantial obstacles. As noted by a community doctor, “Many heart failure patients prefer to go directly to tertiary hospitals rather than utilize community healthcare services. They perceive community doctors as less skilled compared to specialists at tertiary hospitals. This distrust leads to lower patient attendance and poor adherence to follow-up appointments.”

### 3.3 Organizational

#### 3.3.1 Facilitators

The study identified that a strong awareness of chronic disease management and extensive experience in managing chronic conditions within the community are significant facilitators. One community manager noted, “Our community is partnering with a major hospital to pilot chronic disease management for chronic heart failure, and we are excited to take on this initiative.” The community has a long history of managing conditions such as hypertension and diabetes effectively. A community doctor said: “I have been engaged in community chronic disease management for more than 10 years and have been successful in managing hypertension and diabetes. I am also confident in managing chronic heart failure.”

#### 3.3.2 Barriers

##### 3.3.2.1 Inadequate medical knowledge and training programs for medical staff

A significant obstacle to successful CHF treatment is the lack of comprehensive medical education and training programs for healthcare providers. Numerous medical personnel have not undergone adequate training in managing CHF. A community nurse noted, “I have limited knowledge and training regarding chronic heart failure.” Lack of training prevents physicians from effectively diagnosing, treating, and managing CHF. A doctor said: “Chronic heart failure management and continuous learning are necessary, but we have relatively few training programs to keep up with new guidelines.”

##### 3.3.2.2 Shortage of medical staff and limited teamwork

Challenges in CHF management are shortages of medical staff and limited teamwork. Ineffective coordination and collaboration within the healthcare team further complicate matters. “There is a lack of cooperation among healthcare team members, resulting in poor scheduling and work coordination.” said a nurse from a community hospital. Additionally, Leadership and governance are problematic. There is often a perception that performance evaluations are unfair or unreasonable.” The lack of effective teamwork and leadership is detrimental to patient care.

##### 3.3.2.3 Few health promotion channels

There are several barriers to effective CHF management, including a lack of diverse health promotion channels. Inadequate avenues for disseminating health information and promoting healthy behaviors can lead to a lack of patient engagement and education. A community health worker stated, “There are very few resources available for promoting health and educating patients about chronic heart failure. Most of our efforts are limited to lectures or pamphlets, which are not sufficient to engage all patients.” Another nurse echoed this concern, saying, “There is a need for more consistent and widespread efforts in order to truly make an impact on patients, as without regular and varied health promotion activities, many of them remain unaware of critical aspects of managing their condition.”

### 3.4 Community

#### 3.4.1 Facilitators

CHF management is significantly enhanced by the substantial number of community health volunteers. A community manager stated, “Many volunteers visit patients’ homes and conduct disease prevention and control activities, such as cardiovascular health education. In addition to volunteering for various initiatives, our community volunteers provide essential knowledge about disease management to residents.”

#### 3.4.2 Barriers

##### 3.4.2.1 Lack of chronic disease management monitoring systems

In the community, chronic diseases are difficult to manage due to a lack of information technology. A senior community nurse said: “In our clinics, the technology we have does not allow us to monitor patient conditions dynamically or provide timely feedback. We don’t have advanced features like one-touch alarms or peer-to-peer online communication that big cities offer.” And lack of early screening and follow-up for CHF in the community. Medical staff from CHCs reported, “The community does not currently screen for chronic heart failure. As a result, many patients do not know they have chronic heart failure until they experience symptoms. In addition, we do not conduct follow-ups after diagnosis and treatment.”

##### 3.4.2.2 Inadequate medical equipment

A significant barrier at the organizational level is the lack of diagnostic and treatment equipment for CHF. A community nurse noted, “We do not have echocardiograms available in CHCs for chronic heart failure patients, and essential tests like the 6-min walk test are not feasible due to risks and other constraints. Furthermore, the community lacks specialized facilities and equipment for cardiac rehabilitation, and health promotion efforts are insufficient.” A community doctor noted, “We do not have dedicated exercise areas or professional cardiac rehabilitation equipment for heart failure patients. Our current exercise equipment is for the general public, and our health promotion mainly relies on volunteers providing door-to-door education. However, our outreach is limited, and our facilities are not fully equipped for comprehensive cardiac rehabilitation.”

##### 3.4.2.3 Lack of information technology (It) auxiliary management platforms

The coverage of health records is inadequate, leaving us unsure of the exact number of CHF patients in the community and making patient care processes unclear. “Problems with the referral system also hinder effective CHF management, “ a nurse from a tertiary hospital explained, “ The referral system from tertiary hospitals to CHCs is inadequate, with time constraints affecting medical personnel. On the other hand, CHCs have a well-established referral system to tertiary hospitals.” Additionally, the poor monitoring system for chronic disease management hinders progress. A nurse pointed out, “Without a robust monitoring system, it’s hard to continue without proper supervision and evaluation.”

### 3.5 Public policy

#### 3.5.1 Facilitators

Policy makers are more willing, with multiple interviewees saying they “advocate for the government to manage chronic heart failure as a chronic disease. Although this project will take some time, our community hopes it will succeed.”

#### 3.5.2 Barriers

##### 3.5.2.1 Lack of policy support and support funding subsidies

China faces several policy-related obstacles. This includes a lack of policy support for older adults with CHF and insufficient financial assistance. A community hospital administrator said: “China does not provide policy support and sufficient subsidies for the management of chronic heart failure, such as integrating chronic heart failure into community chronic disease management.”

##### 3.5.2.2 Lack of CHF guidelines adapted to the local context

The lack of heart failure guidelines adapted to China is a major challenge for effective management of CHF. “On a day-to-day basis, we don’t follow the guidelines exactly because they don’t match the situation in our community,” one doctor said. Another healthcare provider added: “It’s difficult to have guidelines that reflect local realities. Provide standardized care. As a result, treatment outcomes and patient satisfaction may vary. “In the absence of locally adapted guidelines, it is difficult to consistently implement best practices, ultimately affecting the quality of CHF management.

##### 3.5.2.3 Lack of funding subsidies and low medical insurance reimbursement rate

Low medical insurance reimbursement rates also pose a significant barrier. A doctor stated, “There is a limitation to the coverage of medical insurance, particularly in remote areas with lower reimbursement rates. Rural patients also have difficulty accessing medical resources, and drug treatment reimbursement is minimal.”

## 4 Discussion

This study explores the current state of community-based CHF management among older adults and identifies the factors that facilitate or hinder this management. The significance of this research lies in its focus on enhancing the quality of CHF care within community settings, particularly given the high prevalence and substantial burden of this condition among the older. By employing the SME, the study provides a comprehensive view of the various factors influencing CHF management at multiple levels—individual, interpersonal, organizational, community, and public policy.

### 4.1 Individual level

Although healthcare workers recognize the benefits of chronic disease management for older CHF patients, they often lack the necessary experience and competence. This underscores the need for targeted training programs. This finding is consistent with Heckman GA’s research, which indicates that while healthcare professionals generally have a good base of medical knowledge, there are significant gaps in specialized knowledge, potentially due to uneven access to professional training. Additionally, our study finds that healthcare professionals exhibit strong motivation to learn more about CHF to become effective educators, similar to the enthusiasm documented in Western contexts (21).

Patients face barriers such as traditional beliefs and low health awareness, including poor dietary habits and inadequate exercise, which hinder the community-based management of CHF. Traditional Chinese diets often contain high levels of salt and fat, contributing to chronic health issues. In China, physical labor is typically regarded as exercise, but this may not meet the recommended levels of physical activity required for CHF patients (22). Additionally, older generations in China may have a stronger inclination towards traditional Chinese medicine and skepticism towards Western medicine, which can promote health to some extent but may also delay treatment for acute or severe conditions. Moreover, delayed medical consultations impede early diagnosis and treatment. Cultural beliefs play a crucial role in shaping health behaviors, perceptions of illness, and interactions with healthcare systems (23). In China, traditional beliefs and practices significantly influence patients' approaches to health and illness, affecting everything from preventive practices to treatment adherence and overall health outcomes. Particularly in the post-COVID-19 era, reduced hospital visits due to fear of infection, combined with the psychological trauma experienced by both patients and healthcare workers, have exacerbated these barriers (24).

### 4.2 Interpersonal level

This study finds that good peer support and a strong social media network can be effective in promoting chronic disease management in older adults with CHF. In recent years, the rise of multimedia platforms like WeChat, Kuaishou, and Douyin has enabled even older and illiterate individuals to learn about CHF and connect with peers who share the same condition, providing mutual support and some degree of spiritual comfort. The study by van Harmelen AL found that peer support played an important role in reducing depression, enhancing patient information to combat illness, and reducing social isolation (25). However, family support was found to be inadequate, including a lack of understanding of the illness and insufficient accompaniment of patients during rehabilitation exercises. Family support significantly impacts patients' illness and recovery. Improving family support can greatly enhance patients’ confidence in combating the disease and improve their quality of life and mental health. Therefore, a family-based management approach can be established to provide better health education outcomes.

This study revealed a paradox: while CHF patients are eager to seek care at CHCs and desire appropriate management services, they lack trust in these institutions, leading to poor adherence. Several factors contribute to this contradiction: the current state of community healthcare in China, the relative inexperience of community healthcare workers compared to those in tertiary hospitals, and their limited experience in handling critical illnesses and emergencies, which may reduce patient compliance. Mayer KH found that CHF patients tend to experience frequent recurrences, which exacerbates their distrust of healthcare professionals (26). Additionally, the lack of trust between doctors and patients significantly affects patient adherence, thereby reducing treatment outcomes. Adisa R's research aligns with these findings, showing that as a result of low patient adherence, CHCs have a more difficult time managing chronic diseases, which makes it more challenging to implement CHF management programs in the community. Training programs for healthcare workers should be implemented to enhance their knowledge and competence in order to address these issues. Patients and healthcare workers can also build trust in CHCs by establishing a communication platform for timely feedback.

### 4.3 Organizational level

The inadequacy of medical knowledge and training programs for healthcare staff is one of the major barriers to effective chronic disease management for older CHF patients. Research indicates that medical professionals frequently do not possess current information and thorough education in handling CHF, hindering their capacity to provide the best possible care (27). A knowledge gap affects patient outcomes when training programs do not address the latest advances in treatment protocols and best practices (28–30). According to a study conducted by BA Popescu Chair, regular training can regularly update medical knowledge and clinical skills, thereby improving healthcare's ability to manage chronic diseases (31). In order to improve CHF management and improve the health of CHF patients, it is particularly important to improve the capabilities of medical staff.

Another challenge in managing CHF in older patients is medical staff shortages and limited teamwork. A shortage of healthcare professionals, increasing the workload of existing staff and leading to a decrease in the quality of care (32). Inefficient use of resources often results from poor teamwork and care coordination combined with inadequate performance appraisals (33). Providing effective care for CHF requires a well-coordinated team, requiring good communication, collaborative decision-making, and strong leadership. Implementing structured performance reviews and improving teamwork among healthcare providers can improve care coordination disadvantages, according to study (34). Providing high-quality care to older patients with CHF requires increased staffing and improved teamwork.

A critical gap in the management of CHF in the community is the lack of effective health education channels. Multiple channels of health education can increase awareness and understanding of chronic diseases among older adults (35). Utilizing multimedia platforms such as TikTok, WeChat, and Kuaishou for video content and telephone follow-ups with personalized home health education can greatly enhance disease awareness and patient compliance (36). Especially during epidemics or infectious disease outbreaks, online health education and follow-up are particularly important. The healthcare community should leverage IT platforms for collecting and analyzing health data, providing feedback, and educating patients (37). Modern remote monitoring tools and timely two-way feedback enable patients to promptly discover and improve their own deficiencies, thereby significantly improving the quality of life of CHF patients (37). These methods not only enhance patient compliance but also help reduce readmission rates and overall healthcare costs.

### 4.4 Community level

Lack of early screening and regular follow-up services is another issue facing the community. The early diagnosis and management of CHF depend on early screening. Patients with timely detection have a better chance of surviving and reducing treatment costs (38). In a study by Lund LH, 30% of CHF patients were detected early in CHCs, resulting in a 20% reduction in treatment costs (39). Follow-up visits also provide insights into patient health status by monitoring patient compliance with treatment plans, thus encouraging healthier lifestyles (40).

Community management of CHF is hampered by a lack of necessary diagnostic, treatment and rehabilitation equipment and has a negative impact on patient prognosis and overall health. Effective community CHF management is hampered by a lack of cardiac rehabilitation equipment such as exercise assessment tools, 6-min walk test equipment, exercise cardiopulmonary testing systems, and psycho-emotional assessment tools. Research shows that these devices allow them to exercise more effectively and improve their quality of life. These tools are essential if you want to provide patients with comprehensive diagnosis, treatment, and recovery. Research shows that the rational use of these devices can enable early diagnosis, effective treatment and rehabilitation, enable patients to exercise more effectively, and improve their quality of life (41).

In addition, the lack of an it-assisted management platform affects the management of CHF in the community, which may be influenced by traditional Chinese culture, such as Confucian values that emphasize conservatism and humility, allowing for a lack of innovation. By creating a complete it-assisted management platform that can keep abreast of patient dynamics and give timely feedback, patient disease status can be effectively improved. McKinnon GE's study highlights the importance of an it-supported management platform in reducing communication errors with patients with chronic diseases, thus improving the efficiency of patient-physician interactions (42). Previous studies have shown that mHealth plays a vital role in chronic disease management by providing patients with information resources, risk assessments and reminder services. mHealth can also remotely monitor patients' conditions and exchange data within the healthcare system. In the Chinese community, mHealth has been found to improve the self-management of cardiovascular patients (43, 44).

Chronic disease management is hampered by the lack of a comprehensive electronic health record. According to a study by Kariotis TC, hospitals with electronic health records are better equipped to understand patient information and thus provide high-quality care, compared to hospitals without electronic health records (45). Studies by Uslu A have shown that electronic health records facilitate timely access to patient data and enhance clinical decision-making in chronic diseases management (46). There is a need for the development of an electronic health record system and its full implementation to enhance surveillance to support effective chronic disease management and improve the health status of patients.

In our study, we found that there is a lack of a robust two-way referral system between CHCs and tertiary hospitals. In China, there is an appropriate referral system for patients to be transferred from CHCs to tertiary hospitals, but not vice versa. When a patient is discharged from a tertiary hospital, the disease information is not referred to the patient's community hospital, which prevents CHCs from keeping up to date with the patient's information, thus making follow-up visits impossible (47). The Ferry study found that continuous post-discharge monitoring plays an important role in reducing costs, mortality and readmission rates, and the referral system is an important part of the continuous monitoring and supervision of patients that can be done in CHCs, which confirms the findings of this study. Therefore tertiary hospitals providing detailed treatment plans and follow-up strategies to CHCs is important in improving the management of CHF (48).

### 4.5 Policy level

Currently, there is a lack of appropriate policy support for community-based chronic disease management of older CHF patients in China. Particularly in terms of localised community-based CHF chronic disease-related policy support and appropriate financial support, current policies often fail to meet the special needs of this group. Policies based on frameworks developed in the current Chinese healthcare setting may not adequately address the needs of CHF patients. Community-based chronic disease management in China primarily incorporates hypertension, diabetes, and mental illness, and does not take CHF patients into account and, there is very little economic support for CHF. As a result, some policies cannot be effectively applied to the chronic disease management of older CHF patients (49).

Despite a strong willingness on the part of Chinese policy makers to develop and update guidelines, there is still a lack of guidelines adapted to the Chinese context. Current guidelines for chronic disease management of CHF tend to rely on models developed by European or American heart organizations that are not fully applicable to the current situation in China, such as cost containment, community and patient conditions, and other local issues (1). He WM's study highlighted that Chinese heart failure guidelines are inadequate in terms of “stakeholder involvement,” “applicability,” “editorial independence,” and particularly in addressing “patient perceptions and preferences” and ensuring that “guidelines have been piloted with target users” (50). This gap in guidelines makes implementation challenging. Therefore, it is crucial to develop guidelines suited to China's national conditions for managing CHF to improve clinical outcomes for CHF patients (51).

Additionally, low medical reimbursement rates remain a significant barrier. Despite universal medical insurance in China, inadequate coverage and reimbursement rates continue to affect patients' access to care, particularly in rural areas where reimbursement rates are low and access to quality medical resources is limited (52, 53). Policymakers should consider patients' realities more thoroughly and increase reimbursement rates for CHF, especially for rural patients.

### 4.6 Recommendations for improving community-based CHF management

1. Improve the knowledge and skills of CHCs workers in managing CHF and implement specialized training programs and regular continuing education.
2. Develop a comprehensive IT platform to provide patients and their families with tailored health education to increase family and social support for CHF patients.
3. Establish an IT-assisted management platform and develop a unified two-way referral platform to promote seamless information exchange between CHCs and tertiary hospitals, especially the referral situation of patients into CHCs after being discharged from tertiary hospitals.
4. Equip CHCs with essential cardiac rehabilitation devices, such as exercise assessment tools and heart rate monitors, and improve electronic health record systems.
5. Promote Policy Support Update CHF management guidelines to align with local conditions and increase reimbursement rates, particularly in rural areas, to improve care and support.

## 5 Conclusion

This study provides facilitative and hindering factors for community management of geriatric CHF at different levels. The findings suggest that we should improve the general public's awareness of CHF disease and improve the current status of community healthcare, establish better hospital-community bidirectional referrals, develop policies related to chronic disease management of geriatric CHF, improve CHF chronic disease management guidelines and health care insurance systems in line with national conditions, and develop coping strategies for chronic disease management in special times such as epidemics are important factors in promoting the standardization of community management of geriatric CHF. In addition, individuals, families, society, health care professionals, and policy makers should work together to promote effective chronic disease management of CHF in the older.

### 5.1 Limitations

This study has several limitations. First, it was conducted in only five communities in Zhejiang Province, which limits the generalizability of the findings to other regions in China. The unique regional characteristics of Zhejiang may not reflect the state of CHF management elsewhere in the country. Second, the study primarily analyzed issues from the perspective of healthcare workers, without including the perspectives of patients, their primary caregivers and ect. Future research should expand the range of participants to capture a more comprehensive view of the situation. Third, the number of interviews conducted was relatively small, and future studies could benefit from a larger sample size. Finally, the use of snowball sampling may introduce bias, as participants who know each other may share similar experiences and opinions, potentially skewing the results.

### 5.2 Clinical relevance and future

This study identified several facilitating and hindering factors affecting CHF management in China, particularly within the community setting. At the individual level, healthcare workers often lack sufficient knowledge and skills, while patients demonstrate low health awareness, exacerbated by limited healthcare access during infectious disease outbreaks. At the interpersonal level, inadequate family support, a lack of patient education programs, strained doctor-patient relationships, and poor patient compliance present significant challenges. Organizational barriers include insufficient diagnostic and treatment equipment, incomplete health record systems, a limited referral system, workforce shortages, and inadequate teamwork among healthcare providers. Community-level issues are marked by a lack of screening and follow-up services, inadequate cardiac rehabilitation equipment, and limited health promotion channels. On the policy front, there is a noticeable lack of tailored guidelines for chronic disease management in older CHF patients and insufficient medical insurance reimbursement, particularly in rural areas.

To address these challenges, it is essential to enhance health education initiatives for residents, provide targeted training for community healthcare workers, and strengthen social support systems, especially family involvement. Additionally, improving treatment equipment in CHCs, optimizing the two-way referral system between tertiary and CHCs, and advancing IT infrastructure are critical. Implementing robust community screening and follow-up programs, equipping facilities with cardiac rehabilitation tools, and developing policies tailored to the chronic disease management needs of the older are vital steps. Lastly, improving the reimbursement system for rural patients will ensure equitable access to necessary healthcare services.

## Data Availability

The raw data supporting the conclusions of this article will be made available by the authors, without undue reservation.
